# On the statistical assessment of classifiers using DNA microarray data

**DOI:** 10.1186/1471-2105-7-387

**Published:** 2006-08-19

**Authors:** N Ancona, R Maglietta, A Piepoli, A D'Addabbo, R Cotugno, M Savino, S Liuni, M Carella, G Pesole, F Perri

**Affiliations:** 1 lstituto di Studi sui Sistemi Intelligenti per I'Automazione – CNR, Via Amendola 122/D-l, 70126 Bari, Italy; 2 Unità Operativa di Gastroenterologia, IRCCS, Servizio di Genetica Medica, IRCCS, "Casa Sollievo della Sofferenza"-Ospedale, Viale Cappuccini, 71013 San Giovanni Rotondo (FG), Italy; 4 Dipartimento di Biochimica e Biologia Molecolare – Universitá di Bari, Via E. Orabona 4, 70126 Bari, Italy; 5 lstituto di Tecnologie Biomediche – Sede di Bari – CNR Via Amendola 122/D, 70126 Bari, Italy

## Abstract

**Background:**

In this paper we present a method for the statistical assessment of cancer predictors which make use of gene expression profiles. The methodology is applied to a new data set of microarray gene expression data collected in Casa Sollievo della Sofferenza Hospital, Foggia – Italy. The data set is made up of normal (22) and tumor (25) specimens extracted from 25 patients affected by colon cancer. We propose to give answers to some questions which are relevant for the automatic diagnosis of cancer such as: Is the size of the available data set sufficient to build accurate classifiers? What is the statistical significance of the associated error rates? In what ways can accuracy be considered dependant on the adopted classification scheme? How many genes are correlated with the pathology and how many are sufficient for an accurate colon cancer classification? The method we propose answers these questions whilst avoiding the potential pitfalls hidden in the analysis and interpretation of microarray data.

**Results:**

We estimate the generalization error, evaluated through the Leave-K-Out Cross Validation error, for three different classification schemes by varying the number of training examples and the number of the genes used. The statistical significance of the error rate is measured by using a permutation test. We provide a statistical analysis in terms of the frequencies of the genes involved in the classification. Using the whole set of genes, we found that the Weighted Voting Algorithm (WVA) classifier learns the distinction between normal and tumor specimens with 25 training examples, providing *e *= 21% (*p *= 0.045) as an error rate. This remains constant even when the number of examples increases. Moreover, Regularized Least Squares (RLS) and Support Vector Machines (SVM) classifiers can learn with only 15 training examples, with an error rate of *e *= 19% (*p *= 0.035) and *e *= 18% (*p *= 0.037) respectively. Moreover, the error rate decreases as the training set size increases, reaching its best performances with 35 training examples. In this case, RLS and SVM have error rates of *e *= 14% (*p *= 0.027) and *e *= 11% (*p *= 0.019). Concerning the number of genes, we found about 6000 genes (*p *< 0.05) correlated with the pathology, resulting from the signal-to-noise statistic. Moreover the performances of RLS and SVM classifiers do not change when 74% of genes is used. They progressively reduce up to *e *= 16% (*p *< 0.05) when only 2 genes are employed. The biological relevance of a set of genes determined by our statistical analysis and the major roles they play in colorectal tumorigenesis is discussed.

**Conclusions:**

The method proposed provides statistically significant answers to precise questions relevant for the diagnosis and prognosis of cancer. We found that, with as few as 15 examples, it is possible to train statistically significant classifiers for colon cancer diagnosis. As for the definition of the number of genes sufficient for a reliable classification of colon cancer, our results suggest that it depends on the accuracy required.

## Background

Gene expression from DNA microarray data offers biologists and pathologists the possibility to deal with the problem of cancer diagnosis and prognosis from a quantitative point of view [1]. Conventional tumor diagnosis consists of the examination of the morphological appearance of tissue specimens by trained pathologists. It is subjective and generally it does not allow the establishing of a unique therapy as tumors with similar histopathological appearances can follow different clinical courses [2]. Gene expression data provide a snapshot of the molecular status of a sample of cells in a given tissue, returning the expression levels of thousands of genes simultaneously. They make it possible to analyze the genes involved in a particular type of cancer [3] as well as the classification of tumor specimens in different categories [4,5]. Although DNA microarray data offer enormous opportunities for the definition and understanding of several pathologies, they hide potential pitfalls in their analysis and interpretation [6,7]. A large number of overoptimistic results have been recently published in the literature regarding the possibility of constructing very accurate prediction rules for cancer from only a few genes. Zhang *et al*. [8] showed that a three gene classification tree had an error rate of 2% in colon cancer diagnosis, and Guyon *et al*. [9] showed that a Support Vector Machine (SVM) trained on only two genes had a zero Leave-One-Out (LOO) error in classifying patients with leukemia.

There exists a twofold explanation for such misleading results. The first one concerns the data. Normally, a typical experiment of cancer classification by gene expression data consists of a few number ℓ of specimens, between 10 and 100 examples, each one of which is composed of a large number *d *(in the order of tens of thousands) of gene expression levels. We know that [10] the VC-dimension of the class of linear indicator functions in ℝ^*d *^is *d *+ 1. This means that the simplest classifier, consisting of a separating hyperplane living in the space of the input specimens, is able to separate *d *+ 1 points independently of their labelling. In the application at hand, where the number of features (gene expression levels) *d *is some order of magnitude greater than ℓ, the possibility of separating perfectly the specimens without errors is implied. This problem, known in machine learning literature as "overfitting", is exactly the kind of problem that should be avoided in order to construct predictors able to *generalize*, i.e. which are able to correctly predict the labels of new specimens.

The second reason concerns the methods of analysis. This can be better illustrated through some examples. It has just been said that the ultimate goal of a learning machine is that of generalizing. How is the generalization error of a predictor measured? What is the statistical significance of such a quantity given that it is measured by using only a few examples? Different methodologies will return very different answers. It is well know that the LOO-error provides an almost unbiased estimate of the generalization error of a predictor [11]. Although the bias of the said estimator is low, it is highly variable [6] and has little statistical significance [12]. On the contrary, the Leave-K-Out Cross Validation (LKOCV) error provides a more significant estimate of the generalization error and it should be used to assess the accuracy of a classifier [12]. One further example concerns the methods that select a subset of genes to work with to reduce the problem of overfitting and for finding informative genetic markers of a particular pathology [8,9]. As Ambroise and McLachlan in [6] have admirably pointed out, such methods should carefully avoid the selection bias problem if reliable estimations of the generalization error of predictors are to be obtained. In the present paper a general methodology for the statistical assessment of prediction rules trained by using gene expression data is described, which can be seen as a natural extension of [13] and [12]. The method answers precise questions relevant to cancer diagnosis, avoiding the potential pitfalls connected to microarray data. In this study a new data set of gene expression data is used which was collected from 25 patients affected by colon cancer in "Casa Sollievo della Sofferenza" (CSS) Hospital, San Giovanni Rotondo (FG), Italy. The first set of questions posed concerns the data set. Is the size of the available data set sufficient to build accurate predictors? In which ways does accuracy depend on the prediction model? What is the statistical significance of the prediction error measured? The second set of questions is about the number of gene expression levels. How many genes are correlated with the pathology? How do the accuracy and the statistical significance of the predictor change with respect to the number of the genes used? How does the adopted feature selection strategy influence the prediction error with respect to a random selection of genes? Answers to these questions were provided by using well established models for the classification of gene expression data. In particular we resorted to Weighted Voting Algorithm (WVA) classifiers [1,14], Regularized Least Squares (RLS) classifiers [15,16] and Support Vector Machine (SVM) classifiers [10]. For the assessment of the statistical significance of the classification errors measured, non parametric permutation tests [17,18] were adopted.

## Results

### Data set description

#### Study population

Twenty-five patients (14 males; mean age: 60 ± 14 years), who underwent colonic resection for colorectal cancer (CRC) at CSS hospital, were prospectively recruited into this study. Two samples from each patient were available, one from colon cancer tissue and one from normal colonic mucosa tissue. The samples had been obtained during the surgery, immediately frozen in liquid nitrogen and then stored at -80°C. All of them were reviewed by the same experienced pathologist to confirm the histological diagnosis. None of the patients suffered from hereditary CRC or had received preoperative chemo-radiotherapy. Informed consent to take part in this study was obtained from all the patients. The study was approved by the Hospital's Ethics Committee.

#### RNA extraction from fresh frozen tissue

Total RNA from 150–200 mg of fresh frozen tissue was isolated by phenol-chloroform extraction (TRIzol Reagent, Invitrogen, Carlsbad, CA) and subsequently purified through column chromatography (RNeasy Mini Kit, Qiagen, Valencia, CA) according to the manufacturer's instructions. RNA integrity was monitored using denaturing agarose gel electrophoresis in 1X MOPS. Three neoplastic samples were discarded from the final analysis since their RNA preparation was suboptimal.

#### Microarray assays

Biotinylated target cRNA was generated from 12 mg as described by the Affymetrix Expression Analysis GeneChip Technical Manual (Affymetrix, Santa Clara, California). Briefly, double-stranded cDNA was synthesized from total RNA using the Superscript Choice System (Invitrogen, Carlsbad, California), a primer containing poly(dT) and a T7 RNA polymerase promoter sequence. In vitro transcription using double-stranded cDNA as a template in the presence of biotinylated UTP and CTP was carried out using BioArray High Yield RNA Transcript Labeling Kit (Enzo Diagnostics, Farmingdale, New York). The resulting biotynilated-cRNA "target" was purified and quantified. Fifteen micrograms of biotinylated cRNA were randomly fragmented to an average size of 50 nucleotides by incubating in 40 mM TRIS-acetate, pH 8.1, 100 mM potassium acetate, and 30 mM magnesium acetate at 94°C for 35 minutes. The fragmented cRNA was hybridized for 16 hours at 45°C on Human Genome U133A GeneChips containing a total of 22,283 probe sets and after stained in a Fluidics station with streptavidin/phycoerythrin, followed by staining through a streptavidin antibody and streptavidin/phycoerythrin. Arrays were scanned on a Genearray scanner G2500A by using standard Affymetrix protocols. Absolute data analysis was performed using the Affymetrix Microarray Suite 5.0 software.

### Algorithms

#### Estimating the number of training examples

We are given a data set *S *= {(**x**_1_, *y*_1_), (**x**_2_, *y*_2_), ..., (**x**_ℓ_, *y*_ℓ_)} composed of ℓ labelled specimens, where **x**_*i *_∈ ℝ^*d *^and *y*_*i *_∈ {-1, 1} for *i *= 1, 2,...,ℓ. Let us suppose we have ℓ_+ _positive and ℓ_- _negative examples, such that ℓ = ℓ_+ _+ ℓ_-_. In order to estimate the minimum number of examples to be used for the training of a classifier with a low error rate and a high statistical significance we used a two-step method: a cross validation procedure for the estimation of the error rate of classifiers trained through a given number of examples, and a permutation test for the assessment of the statistical significance of the classification accuracy obtained. In particular, let *n *be the training set size, with *n *= 1, 2,...,ℓ - 1. For every value of *n*, *s*_1 _pairs (*D*_*n*_, *T*_*k*_) of training and test sets are built by random sampling without replacement into the data set *S*, with *n *and *k *as their respective examples, where ℓ = *n *+ *k*. In the training/test split of the data, the same proportion of positive and negative examples as *S *is preserved. For every random split, a classifier is trained by using the examples in *D*_*n *_and its error rate eni
 MathType@MTEF@5@5@+=feaafiart1ev1aaatCvAUfKttLearuWrP9MDH5MBPbIqV92AaeXatLxBI9gBaebbnrfifHhDYfgasaacH8akY=wiFfYdH8Gipec8Eeeu0xXdbba9frFj0=OqFfea0dXdd9vqai=hGuQ8kuc9pgc9s8qqaq=dirpe0xb9q8qiLsFr0=vr0=vr0dc8meaabaqaciaacaGaaeqabaqabeGadaaakeaacqWGLbqzdaWgaaWcbaGaemOBa42aaSbaaWqaaiabdMgaPbqabaaaleqaaaaa@3123@ is evaluated by testing it on *T*_*k*_. The selection of the parameter on which the classifier depends (*C *for SVM and *λ *for RLS classifiers) is carried out by using the examples in *D*_*n *_only. In particular, the *C *parameter in SVM is selected minimizing the three-fold cross validation error [19] and the *λ *parameter in RLS is selected minimizing the LOO-error. Note that in the case of RLS, the evaluation of the LOO-error requires just one training [16]. This procedure for selecting the parameter ensures that eni
 MathType@MTEF@5@5@+=feaafiart1ev1aaatCvAUfKttLearuWrP9MDH5MBPbIqV92AaeXatLxBI9gBaebbnrfifHhDYfgasaacH8akY=wiFfYdH8Gipec8Eeeu0xXdbba9frFj0=OqFfea0dXdd9vqai=hGuQ8kuc9pgc9s8qqaq=dirpe0xb9q8qiLsFr0=vr0=vr0dc8meaabaqaciaacaGaaeqabaqabeGadaaakeaacqWGLbqzdaWgaaWcbaGaemOBa42aaSbaaWqaaiabdMgaPbqabaaaleqaaaaa@3123@ is unbiased as it does not involve the test set *T*_*k*_. So, for each value of *n*, the average error rate en=1s1∑i=1s1eni
 MathType@MTEF@5@5@+=feaafiart1ev1aaatCvAUfKttLearuWrP9MDH5MBPbIqV92AaeXatLxBI9gBaebbnrfifHhDYfgasaacH8akY=wiFfYdH8Gipec8Eeeu0xXdbba9frFj0=OqFfea0dXdd9vqai=hGuQ8kuc9pgc9s8qqaq=dirpe0xb9q8qiLsFr0=vr0=vr0dc8meaabaqaciaacaGaaeqabaqabeGadaaakeaacqWGLbqzdaWgaaWcbaGaemOBa4gabeaakiabg2da9maalaaabaGaeGymaedabaGaem4Cam3aaSbaaSqaaiabigdaXaqabaaaaOWaaabmaeaacqWGLbqzdaWgaaWcbaGaemOBa42aaSbaaWqaaiabdMgaPbqabaaaleqaaaqaaiabdMgaPjabg2da9iabigdaXaqaaiabdohaZnaaBaaameaacqaIXaqmaeqaaaqdcqGHris5aaaa@4080@ is evaluated. Notice that when *n *= ℓ - 1, the classical procedure for the measurement of the LOO-error which involves *s*_1 _= ℓ training/test pairs (*D*_ℓ-1_, *T*_1_) is used. The second step consists of evaluating, for every *n*, the statistical significance of the error rate *e*_*n*_. In a nutshell, we are interested in measuring to what extent the accuracy observed is due to the existing correlation between gene expression levels **x**_*i *_and class labels *y*_*i*_, and how it is observed by chance because of the high dimensionality of the space where the examples live. In order to assess the statistical significance of the error rate the classical method of hypothesis testing is applied. Let *H*_0 _be the null hypothesis in which it is assumed that the random variables **x **and *y *are independent. To evaluate the *p-value *corresponding to *e*_*n*_, it is necessary to know the probability density function of *e*_*n *_under the null hypothesis. Since this is unknown, a nonparametric permutation test [17] is needed, the latter being a method of estimating the empirical probability density function of any statistic under *H*_0 _from the available data. In the contest of classification, the method consists of a) permuting randomly the labels of the training set; b) training a random classifier on this randomly labelled training set and c) testing the classifier obtained on a test set having correctly labelled examples. The reason for this lies in the circumstance that under the null hypothesis all the training sets generated through label permutations are equally likely to be observed, given that the random variables **x **and *y *are independent. Permutation test technique then allows us to determine the percentage of classifiers trained on randomly labelled data having an error rate less than *e*_*n *_in classifying correctly labelled data. In particular the following steps are carried out. For every random split of *S *in training and test sets (*D*_*n*_, *T*_*k*_), we perform *s*_2 _random permutations of the labels of examples belonging to the training set *D*_*n*_. Let Dnπ
 MathType@MTEF@5@5@+=feaafiart1ev1aaatCvAUfKttLearuWrP9MDH5MBPbIqV92AaeXatLxBI9gBaebbnrfifHhDYfgasaacH8akY=wiFfYdH8Gipec8Eeeu0xXdbba9frFj0=OqFfea0dXdd9vqai=hGuQ8kuc9pgc9s8qqaq=dirpe0xb9q8qiLsFr0=vr0=vr0dc8meaabaqaciaacaGaaeqabaqabeGadaaakeaacqWGebardaqhaaWcbaGaemOBa4gabaacciGae8hWdahaaaaa@3113@ be the training set with randomly permuted labels. For every permutation, a classifier is trained by using Dnπ
 MathType@MTEF@5@5@+=feaafiart1ev1aaatCvAUfKttLearuWrP9MDH5MBPbIqV92AaeXatLxBI9gBaebbnrfifHhDYfgasaacH8akY=wiFfYdH8Gipec8Eeeu0xXdbba9frFj0=OqFfea0dXdd9vqai=hGuQ8kuc9pgc9s8qqaq=dirpe0xb9q8qiLsFr0=vr0=vr0dc8meaabaqaciaacaGaaeqabaqabeGadaaakeaacqWGebardaqhaaWcbaGaemOBa4gabaacciGae8hWdahaaaaa@3113@ and the classifier itself is tested on the test set *T*_*k *_which has correctly labelled examples. Even in such a case, the parameter on which the classifier depends is selected by using only the examples in Dnπ
 MathType@MTEF@5@5@+=feaafiart1ev1aaatCvAUfKttLearuWrP9MDH5MBPbIqV92AaeXatLxBI9gBaebbnrfifHhDYfgasaacH8akY=wiFfYdH8Gipec8Eeeu0xXdbba9frFj0=OqFfea0dXdd9vqai=hGuQ8kuc9pgc9s8qqaq=dirpe0xb9q8qiLsFr0=vr0=vr0dc8meaabaqaciaacaGaaeqabaqabeGadaaakeaacqWGebardaqhaaWcbaGaemOBa4gabaacciGae8hWdahaaaaa@3113@. Let us indicate with eni,j
 MathType@MTEF@5@5@+=feaafiart1ev1aaatCvAUfKttLearuWrP9MDH5MBPbIqV92AaeXatLxBI9gBaebbnrfifHhDYfgasaacH8akY=wiFfYdH8Gipec8Eeeu0xXdbba9frFj0=OqFfea0dXdd9vqai=hGuQ8kuc9pgc9s8qqaq=dirpe0xb9q8qiLsFr0=vr0=vr0dc8meaabaqaciaacaGaaeqabaqabeGadaaakeaacqWGLbqzdaWgaaWcbaGaemOBa42aaSbaaWqaaiabdMgaPjabcYcaSiabdQgaQbqabaaaleqaaaaa@3360@ the error rate of the random classifier trained on *n *examples in the *i*-th cross validation and in the *j*-th random permutation. Then the empirical probability density function of the error rate under the null hypothesis is:

pn(e)=1s1s2∑i=1s1∑j=1s2δ(e−eni,j)     (1)
 MathType@MTEF@5@5@+=feaafiart1ev1aaatCvAUfKttLearuWrP9MDH5MBPbIqV92AaeXatLxBI9gBaebbnrfifHhDYfgasaacH8akY=wiFfYdH8Gipec8Eeeu0xXdbba9frFj0=OqFfea0dXdd9vqai=hGuQ8kuc9pgc9s8qqaq=dirpe0xb9q8qiLsFr0=vr0=vr0dc8meaabaqaciaacaGaaeqabaqabeGadaaakeaacqWGWbaCdaWgaaWcbaGaemOBa4gabeaakiabcIcaOiabdwgaLjabcMcaPiabg2da9maalaaabaGaeGymaedabaGaem4Cam3aaSbaaSqaaiabigdaXaqabaGccqWGZbWCdaWgaaWcbaGaeGOmaidabeaaaaGcdaaeWbqaamaaqahabaacciGae8hTdqMaeiikaGIaemyzauMaeyOeI0Iaemyzau2aaSbaaSqaaiabd6gaUnaaBaaameaacqWGPbqAcqGGSaalcqWGQbGAaeqaaaWcbeaakiabcMcaPaWcbaGaemOAaOMaeyypa0JaeGymaedabaGaem4Cam3aaSbaaWqaaiabikdaYaqabaaaniabggHiLdaaleaacqWGPbqAcqGH9aqpcqaIXaqmaeaacqWGZbWCdaWgaaadbaGaeGymaedabeaaa0GaeyyeIuoakiaaxMaacaWLjaWaaeWaceaacqaIXaqmaiaawIcacaGLPaaaaaa@5A4E@

composed of a sum of delta functions centered on the errors measured. The statistical significance (*p-value*) of the error rate *e*_*n *_is given by the percentage of error rates smaller than *e*_*n*_.

#### Estimating the number of genes

The procedure described in the previous section makes it possible to determine the number *n *of training examples to use for building, in principle, an accurate and statistically significant classifier. This section is focused instead on the following problems. How many genes are needed to classify a new specimen? What is the statistical significance of the error rate of a classifier trained by using *n *examples, each of which composed of a subset of *g *genes? In order to answer these questions a methodology is used similar to the one described in the previous section, with the main difference being that this time the specimens are composed of subsets of *g *genes. In particular, for every *g *= 1,2,...,*d*, where *d *is the total number of genes available, *s*_1 _pairs (*D*_*n*_, *T*_ℓ-*n*_) of training and test sets are built by random sampling without replacement into the data set *S*, with *n *and ℓ - *n *examples respectively. Also in this case, the same proportion of positive and negative examples as in *S *is preserved. It should be noted that here the number of training and test examples is constant. The training set is employed to rank the genes according to the value of the statistic [1]:

TS2N(j)=μ+(j)−μ−(j)σ+(j)+σ−(j)j=1,2,...,d     (2)
 MathType@MTEF@5@5@+=feaafiart1ev1aaatCvAUfKttLearuWrP9MDH5MBPbIqV92AaeXatLxBI9gBaebbnrfifHhDYfgasaacH8akY=wiFfYdH8Gipec8Eeeu0xXdbba9frFj0=OqFfea0dXdd9vqai=hGuQ8kuc9pgc9s8qqaq=dirpe0xb9q8qiLsFr0=vr0=vr0dc8meaabaqaciaacaGaaeqabaqabeGadaaakeaafaqabeqacaaabaGaemivaq1aaSbaaSqaaiabdofatjabikdaYiabd6eaobqabaGccqGGOaakcqWGQbGAcqGGPaqkcqGH9aqpdaWcaaqaaGGaciab=X7aTnaaBaaaleaacqGHRaWkaeqaaOGaeiikaGIaemOAaOMaeiykaKIaeyOeI0Iae8hVd02aaSbaaSqaaiab=jHiTaqabaGccqGGOaakcqWGQbGAcqGGPaqkaeaacqWFdpWCdaWgaaWcbaGaey4kaScabeaakiabcIcaOiabdQgaQjabcMcaPiabgUcaRiab=n8aZnaaBaaaleaacqWFsislaeqaaOGaeiikaGIaemOAaOMaeiykaKcaaaqaaiabdQgaQjabg2da9iabigdaXiabcYcaSiabikdaYiabcYcaSiabc6caUiabc6caUiabc6caUiabcYcaSiabdsgaKbaacaWLjaGaaCzcamaabmGabaGaeGOmaidacaGLOaGaayzkaaaaaa@5D8B@

where *j *is the gene index. (*μ*_+ _(*j*), *σ*_+ _(*j*)) and (μ_- _(*j*), *σ*_- _(*j*)) are the mean and the standard deviation of the expression levels of the *j*-th gene in the positive and negative examples respectively, belonging to the current training set. By using the gene list thus sorted, reduced training and test sets (D˜
 MathType@MTEF@5@5@+=feaafiart1ev1aaatCvAUfKttLearuWrP9MDH5MBPbIqV92AaeXatLxBI9gBaebbnrfifHhDYfgasaacH8akY=wiFfYdH8Gipec8Eeeu0xXdbba9frFj0=OqFfea0dXdd9vqai=hGuQ8kuc9pgc9s8qqaq=dirpe0xb9q8qiLsFr0=vr0=vr0dc8meaabaqaciaacaGaaeqabaqabeGadaaakeaacuWGebargaacaaaa@2DCC@_*n*_, T˜
 MathType@MTEF@5@5@+=feaafiart1ev1aaatCvAUfKttLearuWrP9MDH5MBPbIqV92AaeXatLxBI9gBaebbnrfifHhDYfgasaacH8akY=wiFfYdH8Gipec8Eeeu0xXdbba9frFj0=OqFfea0dXdd9vqai=hGuQ8kuc9pgc9s8qqaq=dirpe0xb9q8qiLsFr0=vr0=vr0dc8meaabaqaciaacaGaaeqabaqabeGadaaakeaacuWGubavgaacaaaa@2DEC@_ℓ-*n*_) containing the same examples as the current training and test sets are built, each of which is composed of the *g *genes that are most correlated with the class labels. In particular, each example in the reduced training and test sets contains the expression levels of the first *g*/2 and of the last *g*/2 genes in the list. Such a gene selection strategy provides better results than those provided by ranking the genes according to the absolute value of (2) as reported also in [1,14]. For every random split, a classifier is trained by using those examples in D˜
 MathType@MTEF@5@5@+=feaafiart1ev1aaatCvAUfKttLearuWrP9MDH5MBPbIqV92AaeXatLxBI9gBaebbnrfifHhDYfgasaacH8akY=wiFfYdH8Gipec8Eeeu0xXdbba9frFj0=OqFfea0dXdd9vqai=hGuQ8kuc9pgc9s8qqaq=dirpe0xb9q8qiLsFr0=vr0=vr0dc8meaabaqaciaacaGaaeqabaqabeGadaaakeaacuWGebargaacaaaa@2DCC@_*n *_having *g *components, and its error rate egi
 MathType@MTEF@5@5@+=feaafiart1ev1aaatCvAUfKttLearuWrP9MDH5MBPbIqV92AaeXatLxBI9gBaebbnrfifHhDYfgasaacH8akY=wiFfYdH8Gipec8Eeeu0xXdbba9frFj0=OqFfea0dXdd9vqai=hGuQ8kuc9pgc9s8qqaq=dirpe0xb9q8qiLsFr0=vr0=vr0dc8meaabaqaciaacaGaaeqabaqabeGadaaakeaacqWGLbqzdaWgaaWcbaGaem4zaC2aaSbaaWqaaiabdMgaPbqabaaaleqaaaaa@3115@ is evaluated by testing it on T˜
 MathType@MTEF@5@5@+=feaafiart1ev1aaatCvAUfKttLearuWrP9MDH5MBPbIqV92AaeXatLxBI9gBaebbnrfifHhDYfgasaacH8akY=wiFfYdH8Gipec8Eeeu0xXdbba9frFj0=OqFfea0dXdd9vqai=hGuQ8kuc9pgc9s8qqaq=dirpe0xb9q8qiLsFr0=vr0=vr0dc8meaabaqaciaacaGaaeqabaqabeGadaaakeaacuWGubavgaacaaaa@2DEC@_ℓ-*n *_having examples with *g *components too. Then, for every value of *g*, we evaluate the average error rate eg=1s1∑i=1s1egi
 MathType@MTEF@5@5@+=feaafiart1ev1aaatCvAUfKttLearuWrP9MDH5MBPbIqV92AaeXatLxBI9gBaebbnrfifHhDYfgasaacH8akY=wiFfYdH8Gipec8Eeeu0xXdbba9frFj0=OqFfea0dXdd9vqai=hGuQ8kuc9pgc9s8qqaq=dirpe0xb9q8qiLsFr0=vr0=vr0dc8meaabaqaciaacaGaaeqabaqabeGadaaakeaacqWGLbqzdaWgaaWcbaGaem4zaCgabeaakiabg2da9maalaaabaGaeGymaedabaGaem4Cam3aaSbaaSqaaiabigdaXaqabaaaaOWaaabmaeaacqWGLbqzdaWgaaWcbaGaem4zaC2aaSbaaWqaaiabdMgaPbqabaaaleqaaaqaaiabdMgaPjabg2da9iabigdaXaqaaiabdohaZnaaBaaameaacqaIXaqmaeqaaaqdcqGHris5aaaa@4064@. Two observations should be made. The first is that the procedure of gene ranking involves the examples in the training set only. That is to say, for each iteration the set of *g *genes is determined on the basis of the training examples only. The test set is thus out of the selection process. This makes the estimated error rate selection bias free [6]. The second is that, in general, after each cross validation the list of the *g *selected genes changes.

The second step of the procedure consists in evaluating, for every *g*, the statistical significance of the error rate *e*_*g*_. For this purpose, for every random split of *S*, *s*_2 _random permutations of the labels of examples in the reduced training set D˜
 MathType@MTEF@5@5@+=feaafiart1ev1aaatCvAUfKttLearuWrP9MDH5MBPbIqV92AaeXatLxBI9gBaebbnrfifHhDYfgasaacH8akY=wiFfYdH8Gipec8Eeeu0xXdbba9frFj0=OqFfea0dXdd9vqai=hGuQ8kuc9pgc9s8qqaq=dirpe0xb9q8qiLsFr0=vr0=vr0dc8meaabaqaciaacaGaaeqabaqabeGadaaakeaacuWGebargaacaaaa@2DCC@_*n *_are performed. Let D˜nπ
 MathType@MTEF@5@5@+=feaafiart1ev1aaatCvAUfKttLearuWrP9MDH5MBPbIqV92AaeXatLxBI9gBaebbnrfifHhDYfgasaacH8akY=wiFfYdH8Gipec8Eeeu0xXdbba9frFj0=OqFfea0dXdd9vqai=hGuQ8kuc9pgc9s8qqaq=dirpe0xb9q8qiLsFr0=vr0=vr0dc8meaabaqaciaacaGaaeqabaqabeGadaaakeaacuWGebargaacamaaDaaaleaacqWGUbGBaeaaiiGacqWFapaCaaaaaa@3122@ be the training set with randomly permuted labels. For every permutation, a random classifier is trained by using D˜nπ
 MathType@MTEF@5@5@+=feaafiart1ev1aaatCvAUfKttLearuWrP9MDH5MBPbIqV92AaeXatLxBI9gBaebbnrfifHhDYfgasaacH8akY=wiFfYdH8Gipec8Eeeu0xXdbba9frFj0=OqFfea0dXdd9vqai=hGuQ8kuc9pgc9s8qqaq=dirpe0xb9q8qiLsFr0=vr0=vr0dc8meaabaqaciaacaGaaeqabaqabeGadaaakeaacuWGebargaacamaaDaaaleaacqWGUbGBaeaaiiGacqWFapaCaaaaaa@3122@ and the classifier is tested on the reduced test set T˜
 MathType@MTEF@5@5@+=feaafiart1ev1aaatCvAUfKttLearuWrP9MDH5MBPbIqV92AaeXatLxBI9gBaebbnrfifHhDYfgasaacH8akY=wiFfYdH8Gipec8Eeeu0xXdbba9frFj0=OqFfea0dXdd9vqai=hGuQ8kuc9pgc9s8qqaq=dirpe0xb9q8qiLsFr0=vr0=vr0dc8meaabaqaciaacaGaaeqabaqabeGadaaakeaacuWGubavgaacaaaa@2DEC@_ℓ-*n *_having correctly labelled examples. Let egi,j
 MathType@MTEF@5@5@+=feaafiart1ev1aaatCvAUfKttLearuWrP9MDH5MBPbIqV92AaeXatLxBI9gBaebbnrfifHhDYfgasaacH8akY=wiFfYdH8Gipec8Eeeu0xXdbba9frFj0=OqFfea0dXdd9vqai=hGuQ8kuc9pgc9s8qqaq=dirpe0xb9q8qiLsFr0=vr0=vr0dc8meaabaqaciaacaGaaeqabaqabeGadaaakeaacqWGLbqzdaWgaaWcbaGaem4zaC2aaSbaaWqaaiabdMgaPjabcYcaSiabdQgaQbqabaaaleqaaaaa@3352@ be the error rate of the random classifier trained on D˜nπ
 MathType@MTEF@5@5@+=feaafiart1ev1aaatCvAUfKttLearuWrP9MDH5MBPbIqV92AaeXatLxBI9gBaebbnrfifHhDYfgasaacH8akY=wiFfYdH8Gipec8Eeeu0xXdbba9frFj0=OqFfea0dXdd9vqai=hGuQ8kuc9pgc9s8qqaq=dirpe0xb9q8qiLsFr0=vr0=vr0dc8meaabaqaciaacaGaaeqabaqabeGadaaakeaacuWGebargaacamaaDaaaleaacqWGUbGBaeaaiiGacqWFapaCaaaaaa@3122@ in the *i*-th cross validation and in the *j*-th random permutation. Then the empirical probability density function of the error rate under the null hypothesis is:

pg(e)=1s1s2∑i=1s1∑j=1s2δ(e−egi,j)     (3)
 MathType@MTEF@5@5@+=feaafiart1ev1aaatCvAUfKttLearuWrP9MDH5MBPbIqV92AaeXatLxBI9gBaebbnrfifHhDYfgasaacH8akY=wiFfYdH8Gipec8Eeeu0xXdbba9frFj0=OqFfea0dXdd9vqai=hGuQ8kuc9pgc9s8qqaq=dirpe0xb9q8qiLsFr0=vr0=vr0dc8meaabaqaciaacaGaaeqabaqabeGadaaakeaacqWGWbaCdaWgaaWcbaGaem4zaCgabeaakiabcIcaOiabdwgaLjabcMcaPiabg2da9maalaaabaGaeGymaedabaGaem4Cam3aaSbaaSqaaiabigdaXaqabaGccqWGZbWCdaWgaaWcbaGaeGOmaidabeaaaaGcdaaeWbqaamaaqahabaacciGae8hTdqMaeiikaGIaemyzauMaeyOeI0Iaemyzau2aaSbaaSqaaiabdEgaNnaaBaaameaacqWGPbqAcqGGSaalcqWGQbGAaeqaaaWcbeaakiabcMcaPaWcbaGaemOAaOMaeyypa0JaeGymaedabaGaem4Cam3aaSbaaWqaaiabikdaYaqabaaaniabggHiLdaaleaacqWGPbqAcqGH9aqpcqaIXaqmaeaacqWGZbWCdaWgaaadbaGaeGymaedabeaaa0GaeyyeIuoakiaaxMaacaWLjaWaaeWaceaacqaIZaWmaiaawIcacaGLPaaaaaa@5A36@

composed of a sum of delta functions centered on the errors measured. The statistical significance (*p-value*) of the error rate *e*_*g *_is given by the percentage of error rates smaller than *e*_*g*_.

#### Frequency assessment of the genes selected

It has been stated that the list of *g *genes selected in each cross validation changes because the selection of *n *examples from the data set *S *is random. Nevertheless, since the statistic (2) assigns high scores in absolute value to the genes most correlated with the class labels, the most informative genes are expected to appear in the first/last positions of the list, irrespective of the *n *examples used for evaluating the *T*_*S2N *_statistic. Therefore the frequency *f*_*j *_of appearance of gene *j *in the lists of the genes selected during the cross validation procedure can be used as a measure of the importance of gene *j *in the problem at hand. *f*_*j *_is given by the ratio between the number of appearances of the gene *j *in the top *g *positions and the number *s*_1 _of cross validations. To assess the statistical significance of *f*_*j*_, it is necessary to resort to the permutation test. In particular, *s*_1 _random drawings of *n *examples from *S *are performed and for each one of them *s*_2 _random permutations of the labels of the *n *examples are carried out. For each random permutation of the labels, the genes are sorted according to the values of the statistic (2). The *p-value *associated to *f*_*j *_is given by the frequency of the gene *j *in the top *g *positions in the *s*_1 _× *s*_2 _random permutations of the labels.

### Testing

In this section we try to answer the numerous questions previously raised, showing the results of the methods described as applied to our colon cancer data set. Irrespective of the classifier adopted, the genes are appropriately normalized to have zero mean and unit variance. In particular, for each training and test pair with *n *and ℓ-*n *examples respectively, the *n *training examples are employed to compute the mean and variance of each gene and these parameters are used to normalize the genes in both training and test set. Moreover, linear kernels in RLS and SVM classifiers are used.

#### Training set size

**Figure 1:**
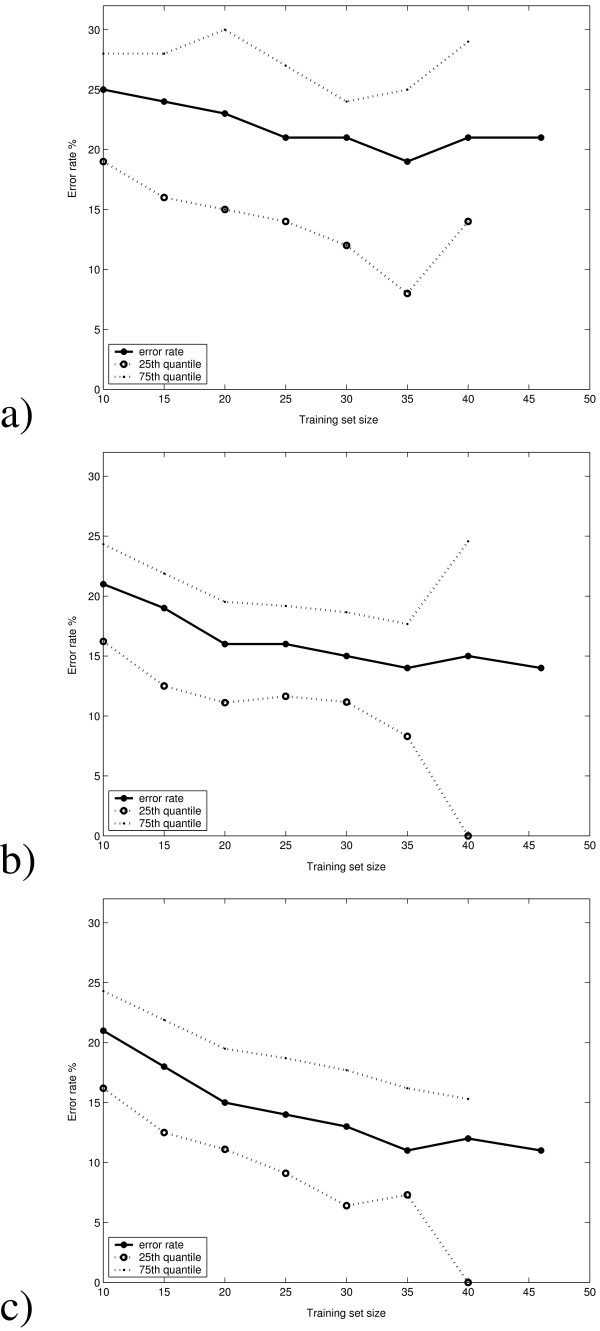
Error rate of a) WVA, b) RLS and c) SVM classifiers varying the training set size.

**Figure 2:**
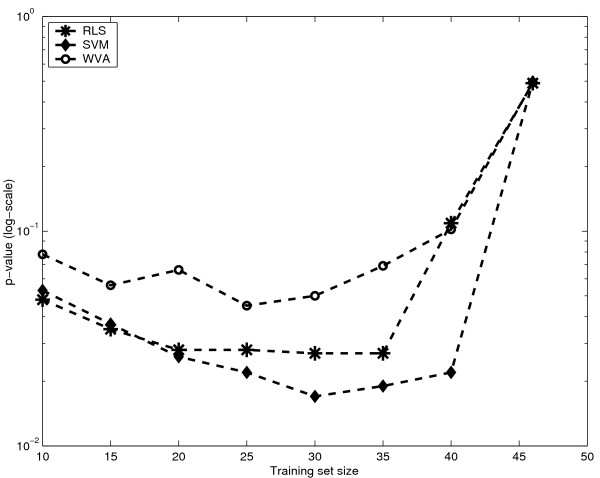
Estimated statistical significance for different training set sizes using WVA, RLS and SVM classifiers.

The first question posed concerns the data set size. How many examples are sufficient for an accurate classification of microarray data of colon cancer? The answer depends, of course, on the classification model adopted. Table 1 shows the error rate *e *and the *p-value p *of three classification schemes, obtained by varying the number of training examples. The error values were estimated performing *s*_1 _= 500 cross validations and *s*_2 _= 500 random permutations of the labels. WVA reaches its minimum error rate of *e *= 19% with *n *= 35 examples, but this estimate has a poor statistical significance (*p *> 5%). The best performance of this model on our data set is reached with *n *= 25 training examples, providing an error rate of *e *= 21% (*p *= 0.045). This table shows that WVA has a limited learning ability, because the error rate does not decrease significantly as the number of training examples is increased (see fig. 1a). RLS and SVM classifiers show a different behavior. Both methods provide classifiers with error rates of *e *≤ 19% (*p *< 5%) with only a few training examples, and their ability of separating tumor from normal specimens improves as the number of training examples increases. The best performances of these classifiers are obtained with *n *= 35 examples. Moreover, the error rate does not improve by increasing the number of training examples, suggesting that *n *= 35 is the optimal number of examples to use for the training of accurate RLS or SVM classifiers (see fig. 1b and 1c). The behavior of the statistical significance of the three classifiers odopted as a function of the training set size is shown in figure 2. As the picture shows, the LOO error exhibits poor statistical significance. Such evidence, reported in [12] as well, seems counter-intuitive if associated to its having been obtained by using the maximum training set size. This is immediately evident if we associate it to the test set size. In the LOO error procedure, the test set is made up of a single example and the likelihood that a random classifier can correctly classify the test example by chance is high. The likelihood decreases as the test set size increases. Having the same the number of training examples, RLS and SVM classifiers show comparable p-values which are always smaller than those of WVA. It should be noted that in all the classification schemes, the LOO error (last row in table 1), in spite of its poor statistical significance, shows values which are comparable to the ones of the LKOCV error when *n *is 30 or 35. This means that the LOO error provides a good estimate of the generalization error of a learning machine [11] and it can be used as a valid alternative to LKOCV error to compare the performances of different classification rules. This aspect is relevant for RLS classifiers which require just one training for the evaluation of the LOO error [16]. Moreover, our results coincide with the ones described in [12] where it is shown that 10–20 examples suffice for the training of classification rules with a statistically significant error rate.

#### Number of genes

**Figure 3:**
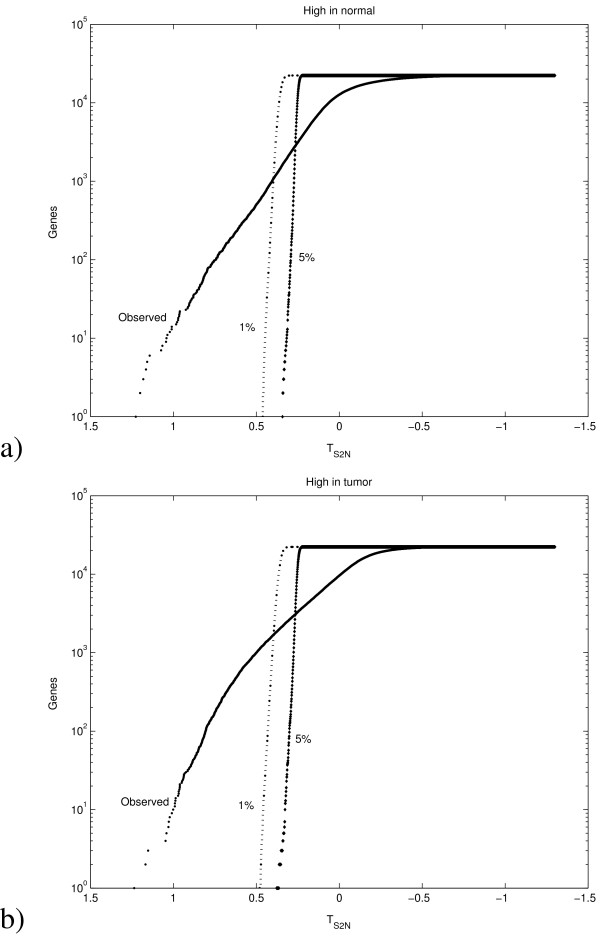
Number of genes more highly expressed in a) normal and b) tumor tissues determined in the actual data set (observed curve) and in data sets with randomly permuted class labels (1% and 5% curves) for different values of the *T*_*S2N *_statistics.

The second question concerns the number of genes. How many genes are sufficient for an accurate classification of gene expression data of colon cancer? In order to be able to answer this question, we applied the method described in the section Algorithms. First of all, the number of genes differentially expressed in our data set, i.e. the ones having a statistically significant value of the statistics (2) had to be determined. To do this, we evaluated (2) on the actual data set and determined the number of genes having a value of the statistics greater than a given threshold. The denoted curve "observed" in figure 3 depicts the number of genes as a function of the statistics *T*_*S2N *_in the actual data set. Every point (*x*, *y*) of the curve represents the number *y *of genes *g *such that *T*_*S2N*_(*g*) ≥ *x*. The same procedure was applied on data sets with randomly permuted class labels. Every point (*x*, *y*) of the curve denoted 1% (5%) in figure 3 represents the number *y *of genes *g *having *T*_*S2N*_(*g*) ≥ *x *with *p-value p *≤ 1% (5%). In this analysis we carried out 1000 random permutations of the labels of the whole data set. As shown in the picture (see the point where observed and 5% curves intersect), about 6000 highly expressed genes (*p *< 5%) were found in the two classes: 3000 genes more highly expressed in normal tissues (figure 3a) and 3000 more highly expressed in tumor tissues (figure 3b).

Table 2 shows the error rate *e *and the *p-value p *of three different classifiers, obtained by varying the number of the genes used. We used *n *= 25 examples for the training of WVA classifiers and *n *= 35 examples for those of RLS and SVM classifiers. We used *s*_1 _= *s*_2 _= 500 in this case as well. It should be noted that WVA always provides error rates with a poor statistical significance, except when the whole set of genes is used. Moreover, the behavior of *e *as a function of *g *shows that this classification model is highly sensible to the noise embedded in the gene expression data. In fact, when the less informative genes are discarded from the classification process, the error rate improves significantly down to 13% with only 32 genes. On the contrary, RLS classifiers show good statistical significance and poor sensibility to the noise because the error rate remains unchanged, as it were, in the whole range of values of *g*. Nevertheless, they are not able to exploit the information embedded in the less informative genes as fully as SVM does. When the whole set of genes is employed, the error rates of RLS and SVM are *e *= 14% (*p *= 0.027) and *e *= 11% (*p *= 0.019) respectively and the errors do not change when the 74% of genes (*g *= 16384) is used. The error rates of the two machines can be compared only when the 37% of genes (*g *= 8192) is used. These results point out that SVM is not influenced by the noise embedded in the data and, most of all, that it is able to exploit the subtle difference between normal and tumor specimens hidden in the less informative genes. Moreover, the results described above show that several cell products are altered in colon cancer and that an accurate classification is possible only by taking into account the expression levels of thousands of genes simultaneously.

#### Frequency analysis of the genes selected

**Figure 4:**
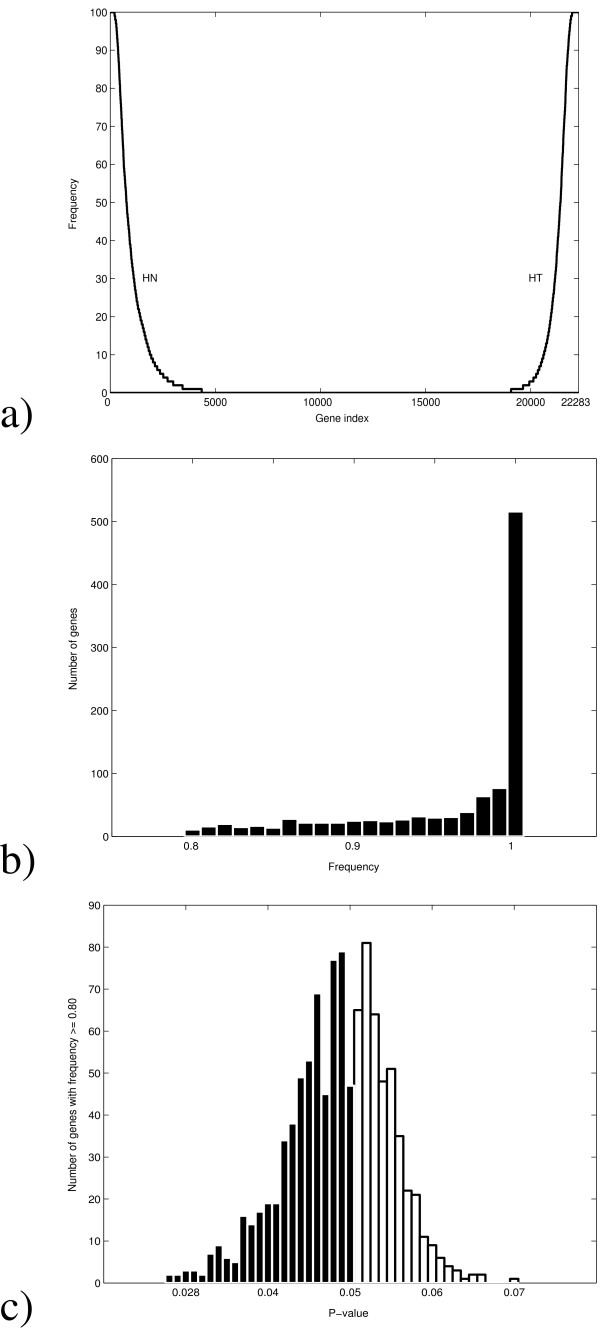
Frequency analysis of the genes selected. a) Frequencies of all the genes in the top *g *= 2048 positions in the sorted gene list. The frequencies of the highly expressed genes in normal and tumor specimens are indicated with HN and HT respectively. b) Number of genes with frequency ≥ 80% and c) the number of genes with a given p-value.

In order to analyze the frequency of appearance *f*_*j *_of the gene *j *= 1, 2,..., *d *in the lists of the genes *g *selected in the cross validation procedure, *s*_1 _= 100 random drawings of *n *= 35 examples from the data set *S *were carried out; for each drawing, the genes were sorted according to the value of the statistic (2). The frequency *f*_*j *_was evaluated by counting the presence of the gene *j *in the top *g *= 2048 positions (the first 1024 and the last 1024) in the lists of the sorted genes. Figure 4a) depicts the frequencies of all the genes available. It can be seen that more than half of the genes do not appear in the top *g *positions of the list. Moreover, 1078 genes were found (467 more highly expressed in normal specimens and 611 in tumor ones) to have a frequency greater than 80% (see figure 4b) and, among these, 516 had a frequency of 100%. Aiming to assess the statistical significance of these frequencies, we performed *s*_2 _= 100 random permutations of the labels of the *n *examples in each random drawing. Figure 4c) depicts the number of genes with *f*_*j *_≥ 80% of which having a given *p-value*. Thanks to this analysis, 647 statistically significant genes (*p *< 0.05) were found.

#### Biological analysis

Among the statistically significant genes, 92 genes differentially expressed between normal tissue and matched tumour tissue, are reported in tables 3 and 4. Most genes have been already shown to be involved in colorectal tumorigenesis. A brief description of 45 genes up- and 47 genes down-regulated in tumour tissue, which could be used as diagnostic biomarkers or targets for therapy, is reported. At least 31 genes of cell cycle have been shown to be up-regulated in our colon cancer specimens. The mitotic checkpoint is an important signalling cascade that arrests the cell cycle in mitosis when even a single chromosome is not properly attached to the mitotic spindle [20]. It has been postulated that defects in the levels of mitotic checkpoint proteins could be responsible for mitotic checkpoint impairment and aneuploidy with disruption of genomic integrity. However, until now, no functionally significant sequence variations of mitotic checkpoint genes has been detected in colorectal cancer [21]. Conversely, we found that 6 genes involved in the mitotic spindle checkpoint (TTK, BUB1, BUB3, CDC20, MAD2L1, and BUB1B) are overexpressed in colon cancer specimens. Very recently, an increased expression of mitotic spindle checkpoint transcripts has been reported in breast cancers with chromosomal instability [22] suggesting that mitotic checkpoint impairment in human tumor cells (and chromosomal instability) could be due to increased levels of mitotic checkpoint proteins rather than mutations in checkpoint genes. In tumour, these changes could occur through altered transcriptional regulation by tumour suppressors or oncogene products. Drugs that specifically and efficiently interfere with mitotic checkpoint signalling could therefore be useful as anticancer agents. Another process which is deeply disorganized in cancer is cell growth with several cellular processes and mechanisms that control cell cycle progression deregulated. In non neoplastic cells, these events are highly conserved due to the existence of conservatory mechanisms and molecules such as cell cycle genes and their products: cyclins, cyclin dependent kinases, Cdk inhibitors (CKI) and extra cellular factors (i.e. growth factors). At least 25 genes of cell cycle progression have been shown to be up-regulated in our colon cancer specimens. They include CDC2, the universal inducer of mitosis, cyclin B and CDC25, which interact with the CDC2 to regulate both G1/S and G2/M transitions (checkpoints) of the cell cycle, and the MCM genes which are required for the entry in S phase and for genome duplication.

Four up-regulated genes involved in the cell cycle progression are of particular interest in colon tumorigenesis: CKS1, CKS2, SKP2, and FOXM1. Both CKS1 and SKP2 are involved in regulation of G1/S transition and in degradation of CDKN1B (p27) a putative gene suppressor. Colorectal tumours with high levels of CKS1 and SKP2 generally exhibit a more aggressive behaviour and are associated with low levels of CDKN1B (p27) and loss of tumor differentiation [23]. Moreover, CKS2 is expressed at significantly higher levels in colorectal tumors with liver metastasis [24]. Apart from their prognostic significance, these genes could also represent optimal targets for gene therapy. Recently, the effect of transfection of Cks1-specific small interfering RNA (siRNA) in human Cks1-overexpressing H358 lung cancer cell lines has been tested: Cks1 siRNA down-regulated Cdc2 kinase activity and induced G2/M arrest. Long-term treatment of Cks1 siRNA induced caspase activation and apoptosis [25]. The FOXM1 gene is critical for G1/S transition and essential for transcription of cell cycle genes such as SKP2 and CKS1 [26]. Other 7 up-regulated genes involved in cell mitosis are STK15, SRPK1 and TOP2A, and SMC4L1, CNAP1, HCAP-G, and KIF4A. All of them have been found overexpressed in some cancer lines and some tumour cells and may represent both prognostic indicators and molecular target for anticancer drugs. STK15 is a critical centrosome-associated kinase-encoding gene overexpressed in multiple human tumour cell types which is involved in the induction of centrosome duplication-distribution abnormalities, chromosomal instability, and aneuploidy in mammalian cells [27]. It could represent an optimal target for chemotherapy. SRPK1 and TOP2A are part of a multisubunit complex, named toposome, containing ATPase/helicase proteins (RNA helicase A and RHII/Gu), HMG protein (SSRP1), and pre-mRNA splicing factors (PRP8 and hnRNP C) which is involved in separating entangled circular chromatin DNA during chromosome segregation. In particular, SRPK1 plays a central role in the pre-mRNA splicing, a critical step in the posttranscriptional regulation of gene expression. Aberrant patterns of pre-mRNA splicing have been established for many human malignancies. Recently, it has been shown that SRPK1 is overexpressed in tumors of the pancreas, breast, and colon and siRNA-mediated down-regulation of SRPK1 in tumour cell lines results in a dose-dependent decrease in proliferative capacity and increase in apoptotic potential [28]. These findings support SRPK1 as a new, potential target for the treatment of cancer.

Finally, SMC4L1, CNAP1, and HCAP-G are components of the condensin complex, which also contains other four subunits: SMC2L1, BRRN1, CAPH, and CAPD2 [29]. KIF4A is proposed to be a motor protein carrying DNA as cargo in condensed chromosomes throughout mitosis interacting with condensin complex [30]. The condensin complex is required for conversion of interphase chromatin into mitotic-like condense chromosomes. Interestingly, CDC2, the universal inducer of mitosis, phosphorylates HCAP-G, CNAP1, and BRRN1, thus activating the condensin complex and chromosome condensation. Among the up-regulated genes in colorectal cancer, we found 14 genes involved in signal transduction (TDGF1 and ENC1), transcription (SOX9, MYC, and HGFR/MET), nuclear transport (NUP62, NUPL1, NUP155, KPNA2, RANBP5, CSE1L/CAS, NTF2, and RANBP1) and cellular transport (SLCO4A1). TDGF1, a growth factor with an EGF-like domain, is over-expressed in breast, cervical, ovarian, gastric, lung, colon, and pancreatic carcinomas in contrast to normal tissues where TDGF1 expression is invariably low or absent. TDGF1 is released or shed from expressing cells and may serve as an accessible marker gene in the early to mid-progressive stages of breast and other cancers [31]. ENC1 is another transduction gene probably involved in differentiation of epithelial cells as well as in cell proliferation. ENC1 is regulated by the beta-catenin/Tcf pathway and up-regulated in colorectal cancer where it may suppress differentiation of colonic cells [32]. SOX9 is a transcription factor and seems to be expressed throughout the intestinal epithelium under the control of the Wnt-pathway. Its function may be to maintain healthy and tumor epithelial cells in undifferentiated state [33]. MYC and HGFR/MET are two well-known oncogenes which activate the transcription of growth-related genes. Overexpression of MYC and HGFR/MET is implicated in the aetiology of a variety of tumours and would serve as an important therapeutic target. Eight genes involved in nucleocytoplasmic transport were up-regulated in colon cancer. Nuclear-cytoplasmic transport, which occurs through special structures called nuclear pores, is an important aspect of normal cell function, and defects in this process have been detected in many different types of cancer cells.

Overproduction of nuclear transport factors such as KPNA2, RANBP5, NTF2, and CSE1L/CAS may disrupt the nuclear import and export machinery leading to loss of nuclear transport of several proliferation activating proteins, transcription factors, oncogene and tumour suppressor gene products and, finally, to cell transformation [34]. One up-regulated gene with transport function has been detected: SLCO4A1/OATP1 belongs to a membrane transport systems superfamily with multiple expression in the liver, kidney, small intestine, and choroid plexus barrier. It acts as a mediator in the sodium-independent transmembrane solute transport and has a strategic position for absorption, distribution and excretion of xenobiotic substances [35]. At least 3 genes involved in apoptosis have been shown to be down-regulated in our colon cancer specimens. FAS and CASP7 are involved in the activation cascade of caspases responsible for apoptosis. Both could be involved in tumour progression and poorer prognosis as shown in urothelial cancer [36]. PDCD4 is a well known tumour suppressor gene involved in apoptosis and inhibition of protein translation. Loss of PDCD4 is associated with tumour progression and prognosis [37] while overexpression of PDCD4 in human colon carcinoma cells is able to suppress tumour progression by inhibiting c-Jun and AP-1 pathways [38]. These findings implicate a potential value of PDCD4 as a molecular target in cancer therapy. Molecular transport and cell metabolism are strongly impaired in cancer cells. Consequently it is not surprising that microarray analysis revealed down-regulation of several genes coding for proteins of transport and metabolism. Loss of carriers profoundly affects the intracellular concentration of solutes such as sodium, potassium, hydrogen, and bicarbonate which are involved in several metabolic pathways. Loss of enzymes which control the most important metabolic pathways have a negative influence on cell physiology and, most importantly, might render cancer cell less sensitive or resistant to anticancer drugs.

Of relevance is the down-regulation of most carbonic anhydrases which control pH homeostasis and modulate the behaviour of cancer cells. In our specimens, several isozymes of carbonic anhydrases (I, II, IV, VII, and XII) were down-regulated implying a pathogenic role in cancer development or progression. Several genes coding for proteins involved in intracellular and cell surface signalling pathways were down-regulated in colon cancer. In our analysis, down-regulation of genes such as MAP2K4, RPS6KA5, MEF2C, SHOC2 produces a serious impairment of the MAPK signalling cascade involved in cell growth and differentiation. Similarly, other down-regulated genes such as PPP2R3A, MUC4, SOCS2 and SMAD2 may contribute to impair Wnt, Erb2, GH, and TGF-beta pathways involved in several cellular processes. NDRG2, EPB41L3, MTUS1 are three down-regulated genes implicated in cell differentiation. They represent three candidate tumour suppressor genes and are often inactivated in tumours [39,41]. Their relevance in colon cancer progression and prognosis is still to be determined. Other three down-regulated genes implicated in negative control of cell growth have been identified by microarray analysis: FAM107A (TU3A), BTG1, and KLF4. TU3A has been found also down regulated in renal cancer cells [42]: even if its molecular function is unknown, it could represent a novel suppressor gene. BTG1 is an antiproliferative protein involved in apoptosis. Its role in colonic carcinogenesis is still to be elucidated. Finally, KLF4, an inhibitor of the cell cycle, has been recently found down-regulated in colonic [43] and gastric cancer. Loss of expression of KLF4 is associated with cancer progression [44].

## Discussion and conclusions

The present paper describes a general methodology for the assessment of the statistical significance of prediction rules trained to classify DNA microarray data. The method, which can be considered a natural extension of the ones proposed in [12,13], provides statistically significant answers to precise questions relevant to the diagnosis and prognosis of cancer. The method has been applied to a new DNA microarray data set collected in Casa Sollievo della Sofferenza Hospital, Foggia – Italy, relative to patients affected by colon cancer. We have found that it is possible to train statistically significant classifiers for colon cancer diagnosis with as few as 15 examples. This result agrees with the one described in [12] and it bears out the empirical observation that tumor morphological distinctions (including disease versus normal classification) are, in general, easier to deal with than those concerning the treatment outcome prediction. In our case, the best classification performance was achieved by training an SVM classifier with 35 examples, which produced an error rate of *e *= 11% (*p *= 0.019). This shows that the size of our data set is sufficient to build statistically significant classifiers for colon cancer diagnosis.

Concerning the problem of determining a sufficient number of genes to be used for an accurate classification of colon cancer, our results suggest that it depends on the accuracy required. In fact, the error rate ranges between *e *= 11% (*p *= 0.025), obtained training SVM classifiers with *g *= 16384 genes, and *e *= 16% (*p *< 0.05) obtained training RLS or SVM classifiers with only *g *= 2 genes. This result indicates that a remarkable number of genes are altered in the pathology and that a lot of them convey useful information for the classification of new specimens. In order to verify such a result, the following experiment was carried out. We trained an SVM classifier with 35 examples each of which composed of 64 genes *randomly *drawn from the set of all the genes available, thus obtaining an error rate of *e *= 23% (*p *= 0.038). This value, although higher than the one obtained by using gene lists ranked with the *T*_*S2N *_statistic (see table 2), indicates that many different sets of 64 genes can be used to build accurate classifiers. The behavior of e as a function of *g *is consistent and has been pointed out by other authors. For example, [45] finds a decreasing behavior of the error rate w.r.t. *g *by analyzing three microarray data sets, with different gene selection criteria. In conclusion, our results indicate that a highly accurate and statistically significant classification of colon specimens is possible even when a small number of genes is employed.

Some conclusions can be drawn concerning the classification models involved in our analysis. WVA classifiers show poor generalization ability and they are greatly influenced by the noise embedded in the microarray data. They rarely provide statistically significant classification performances and, for these reasons, they should not be used as predictors of DNA microarray data. On the contrary, RLS classifiers performances are comparable to those of SVM classifiers, the state-of-the-art supervised learning machines in many application domains, including cancer classification by DNA microarray data [5]. The main advantage of RLS machines in solving a classification problem lies in their employment of a linear system of order equal to either the number of genes or the number of training examples. This property is extremely important and reduces the computational cost of the permutation test because, for a fixed random split of the data, the coefficients of random classifiers are obtained by multiplying a constant matrix with vectors of randomly permuted labels [16]. Moreover, RLS machines allow us to get an exact measure of the LOO error with just one training. For all these reasons and because of their simplicity and low computational complexity, RLS classifiers provide a valuable alternative to SVM classifiers with regard to the problem of cancer classification by gene expression data. Moreover, RLS classifiers show generalization abilities comparable to the ones of SVM classifiers even when the classification of new specimens involves very few gene expression levels. The last consideration concerns the way in which these two classification schemes represent the solution. SVM tends to give sparse solutions in terms of number of training examples and RLS tends to give sparse solutions in terms of number of features used for classifying.

Colorectal cancer is the third most common cancer in men and women and accounts for 11% of all cancer deaths. Whereas the 5-year survival rate is extremely favorable when detected at a localized stage (90%), most colorectal cancers are either locally or distantly invasive at diagnosis, limiting treatment options and lowering survival rates. Clearly, a more comprehensive view of the molecular events associated with colorectal tumorigenesis is needed to identify tumours earlier and to treat colorectal tumours more effectively. Microarray technology has the potential to detect tumour-specific genes which can be used as biomarkers for early diagnosis and specific treatments. Potential uses of this technology include determining who will benefit from chemotherapy, further classifying patients into responders and nonresponders, predicting apoptotic response, developing classifiers to recognize chemosensitive tumors, identifying genes that portend a poor prognosis, revealing genes associated with metastases, predicting the outcome according to clinical stage, and avoiding surgery in patients who would not benefit from resection.

In this study, by means of specific statistical methods, we have found several genes up- and down-regulated in colon cancer which could be used as diagnostic biomarkers or therapeutic targets. Among the up-regulated genes, the most representative are those implicated in mitotic checkpoint signalling cascade and those controlling cell cycle progression. Inhibition of overexpressed genes is potentially useful to control cancer growth. Among the down-regulated genes, the most interesting for their potential therapeutic implication are those of apoptosis, intracellular and cell surface signalling, and cell arrest. Reactivation of their function could be useful to suppress cancer development or progression. A few of these up- and down-regulated genes have not been described in colon cancer yet. Further studies focused on these genes and related transcripts are necessary to better elucidate their pathogenic role in colon cancer disease and their clinical relevance in diagnostics and therapeutics.

## Authors' contributions

NA and FP conceived the study. NA, RM and AD designed the algorithms and conduced the experiments and, together with SL and GP, they evaluated and compared the experimental results. AP, RC, MS and MC were mainly involved in the population study, RNA extraction and the provision of the final DNA microarray data set. All the authors contributed to the drafting of the article. All authors read and approved the final manuscript.

## Figures and Tables

**Table 1:** Error rate *e *and *p-value p *for different training set sizes.

	WVA		RLS		SVM	
*n*	e	*p*	e	*p*	e	*p*

10	25%	0.078	21%	0.048	21%	0.053
15	24%	0.056	19%	0.035	18%	0.037
20	23%	0.066	16%	0.028	15%	0.026
25	21%	0.045	16%	0.028	14%	0.022
30	21%	0.050	15%	0.027	13%	0.017
35	19%	0.069	14%	0.027	11%	0.019
40	21%	0.102	15%	0.109	12%	0.022
46	21%	0.493	14%	0.489	11%	0.495

**Table 2:** Error rate e and *p-value p *of classifiers trained with a fixed number of examples and a different number of genes.

	WVA		RLS		SVM	
*g*	*e*	*p*	*e*	*p*	*e*	*p*

22283	21%	0.045	14%	0.027	11%	0.019
16384	20%	0.065	14%	0.021	11%	0.025
8192	18%	0.073	14%	0.034	14%	0.039
4096	16%	0.116	14%	0.021	14%	0.039
2048	15%	0.168	14%	0.034	14%	0.033
1024	14%	0.216	13%	0.024	13%	0.040
512	13%	0.118	13%	0.028	14%	0.033
256	13%	0.127	13%	0.040	14%	0.025
128	13%	0.139	13%	0.036	14%	0.013
64	13%	0.142	13%	0.036	14%	0.022
32	13%	0.131	13%	0.022	14%	0.031
16	14%	0.242	13%	0.030	14%	0.040
8	15%	0.202	14%	0.029	14%	0.041
4	16%	0.165	14%	0.041	16%	0.031
2	19%	0.213	16%	0.046	16%	0.041

**Table 3:** 45 genes up-regulated in tumoral tissue, comparing normal mucosa to matched tumor colon tissue.

Function	Gene	OMIM	Accession no.	p-value	Gene description
Cell cycle: mitosis (spindle checkpoint)	TTK	604092	NM_003318.1	0.029	Threonine-tyrosine kinase
	BUB1	602452	AF043294.2	0.035	Budding uninhibited by benzimidazoles 1 homolog (yeast)
	BUB3	603719	NM_004725.1	0.037	Budding uninhibited by benzimidazoles 3 homolog (yeast)
	CDC20	603618	NM_001255.1	0.044	Cell division cycle 20
	MAD2L1	602686	NM_002358.2	0.049	MAD2 (mitotic arrest deficient, yeast, homolog) like-1
	BUB1B	602860	NM_001211.2	0.050	Budding uninhibited by benzimidazoles 1 homolog beta (yeast)
Cell cycle: G0/G1 transition	INSIG1	602055	NM_005542.1	0.039	Insulin induced gene 1 (cell division cycle, G0 to G1)
Cell cycle: mitosis (G1/S checkpoint)	CKS2	116901	NM_001827.1	0.047	CDC28 protein kinase regulatory subunit 2
	CKS1B	116900	NM_001826.1	0.046	CDC28 protein kinase regulatory subunit 1B
	SKP2	601436	BG105365	0.050	S-phase kinase-associated protein 2 (p45)
	FOXM1	602341	NM_021953.1	0.045	Forkhead box M1
	MCM4	602638	AA 604621	0.036	Minichromosome maintenance deficient (S. cerevisiae) 4
	MCM3	602693	NM_002388.2	0.048	Minichromosome maintenance deficient (S. cerevisiae) 3
	MCM7	600592	D55716.1	0.048	Minichromosome maintenance deficient 7 (S. cerevisiae)
	MCM2	116945	NM_004526.1	0.049	Minichromosome maintenance deficient (S. cerevisiae) 2
	MCM6	601806	NM_005915.2	0.050	Minichromosome maintenance deficient (S. pombe) 6
Cell cycle: mitosis (G1/S and G2/M checkpoints)	CRKRS		M68520.1	0.039	Cdc2-related kinase, arginine/serine-rich
	CDC2/CDK1	116940	NM_001786.1	0.044	Cell division cycle 2, G1 to S and G2 to M
	CDC25A	116947	NM_001789.1	0.050	Cell division cycle 25A
	CDC25B	116949	NM_021873.1	0.050	Cell division cycle 25B
	CCNA2	123835	NM_001237.1	0.050	Cyclin A2
Cell cycle: mitosis (G2/M checkpoint)	CCNB1	123836	Hs.23960	0.047	Cyclin B1(cell division cycle, G2 to M)
	CCNB2	602755	NM_004701.2	0.047	Cyclin B2 (cell division cycle, G2 to M)
	NEK2	604043	NM_002497.1	0.037	NIMA (never in mitosis gene a)-related kinase 2
Cell cycle: mitosis	STK15	602687	NM_003600.1	0.039	Serine/threonine kinase 6 (chr segregation)
	SRPK1	601939	NM_003137.1	0.046	SFRS protein kinase 1 (chr segregation)
	TOP2A	126430	NM_001067.1	0.050	Topoisomerase (DNA) II alpha (170 kD) (chr segregation)
	KIF4A	300521	NM_012310.2	0.035	Kinesin family member 4A (spindle formation/chr condensation)
	CNAP1	609689	NM.014865	0.046	Chromosome condensation-related SMC-associated protein 1
	SMC4L1		NM_005496.1	0.048	SMC4 structural maintenance of chromosomes 4-like 1 (yeast)
	HCAP-G	606280	NM_022346.1	0.042	Chromosome condensation protein G (chr condensation)
Signal transduction	TDGF1	187395	NM_003212.1	0.048	Teratocarcinoma-derived growth factor 1 (EGF signaling)
	ENC1	605173	NM_003633.1	0.048	Pig 10, ectodermal-neural cortex (WNT//beta-catenin pathway)
Transcription	SOX9	608160	NM_000346.1	0.045	Sex determining region Y-box 9
	MYC	190080	NM_002467.1	0.047	V-myc avian myelocytomatosis viral oncogene homolog
	HGFR/MET	164860	NM_002467.1	0.047	Met proto-oncogene
Transport: intracellular	NUP62	605815	NM_012346.1	0.039	Nucleoporin 62 kD
	NUPL1	607615	NM_007342.1	0.050	Nucleoporin-like 1
	NUP155	606694	NM_004298.1	0.045	Nucleoporin 155 kD (NUP155)
	KPNA2	600685	NM_002266.1	0.045	Karyopherin alpha 2 (RAG cohort 1, importin alpha 1)
	RANBP5	602008	NM_002271.1	0.050	RAN binding protein 5 or karyopherin (importin) beta 3
	CSE1L/CAS	601342	NM_001316	0.050	CSE1 chromosome segregation 1-like (yeast)
	NXT1	605811	NM_005796.1	0.050	Nuclear transport factor 2 (NTF2)
	RANBP1	601180	NM_002882.2	0.048	RAN binding protein 1
Transport	SLCO4A1	605495	NM_016354.1	0.048	Solute carrier family 21 (organic anion transporter)

**Table 4:** 47 genes down-regulated in tumoral tissue, comparing normal mucosa to matched tumor colon tissue.

Function	Gene	OMIM	Accession no.	p-value	Gene description
Apoptosis	PDCD4	608610	NM_014456.1	0.032	Programmed cell death 4 (neoplastic transformation inhibitor)
	FAS	604306	NM_000043.1	0.044	Fas (TNF receptor superfamily, member 6)
	CASP7	601761	NM_001227.1	0.050	Caspase 7, apoptosis-related cysteine protease
Transport	SLC30A10		NM_018713.1	0.036	Solute carrier family 30, member 10 (zinc transport?)
	SLC9A2	600530	AF073299.1	0.041	Solute carrier family 9 (sodium/hydrogen exchanger), member 2
	SLC4A4	603345	AF069510.1	0.041	Solute carrier family 4, sodium bicarbonate cotransporter, member 4
	SLC26A3	126650	NM_000111.1	0.044	Solute carrier family 26, member 3
	SLC26A2	606718	AI025519	0.044	Solute carrier family 26 (sulfate transporter), member 2
	SGK2	607589	NM_016276.1	0.038	Serum glucocorticoid regul. kinase 2 (potassium channel activation)
	KIF5C	604593	NM_004522.1	0.040	Kinesin family member 5C (intracellu-lar transport)
	KIF13B	607350	NM_015254.1	0.046	Kinesin family member 13B (intracel-lular transport)
	VAPA	605703	AF154847.1	0.047	VAMP (vesicle-associated membrane protein)-assoc. protein A,33 kDa
Signalling	MAP2K4	601335	NM_022129.1	0.033	Mitogen-activated protein kinase kinase 4 (MAPK signaling pathway)
	RPS6KA5	603608	AF074393.1	0.040	Ribos. prot. S6 kinase, 90 kDa, polyp. 5(MAPK signalling pathway)
	MEF2C	600662	L08895.1	0.033	MADS box transcr. enhancer factor 2, (MAPK signalling pathway)
	PPP2R3A	604944	NM_002718.1	0.037	Protein phosphatase 2, regulatory sub-unit B, alpha (Wnt signalling)
	PDE9A	602973	NM_002606.1	0.040	Phosphodiesterase 9A (signal transduc-tion)
	PPAP2A	607124	AF014403.1	0.042	Phosphatidic acid phosphatase type 2A (signal transduction)
	MUC4	158372	AJ242547.1	0.044	Mucin 4 (Erb2 signalling pathway)
	DSCR1	602917	AL049369.1	0.045	Down syndrome critical region gene 1 (signal transduction)
	SHOC2	602775	NM_007373.1	0.046	Soc-2 suppressor of clear homolog (MAPK signaling pathway)
	SOCS2	605117	NM_003877.1	0.049	Suppressor of cytokine signaling 2 (GH/IGF1 signaling pathway)
	SMAD2	601366	NM_005901.1	0.049	SMAD, homolog 2 (Drosophila) (TGF-beta_signaling)
Cell-surface signalling	TSPAN7	300096	NM_004615.1	0.036	Tetraspanin 7
	EDG2	602282	NM_001401.1	0.041	Lysophosphatidic acid G-protein-coupled receptor, 2
	TMPRSS2	602060	AF270487.1	0.046	Transmembrane protease, serine 2
	CEACAM7		NM_006890.1	0.047	Carcinoembryonic antigen-related cell adhesion molecule 7
Cell adhesion	DSC2	125645	NM_004949.1	0.045	Desmocollin 2
Cell differentiation	NDRG2	605272	NM_016250.1	0.038	NDRG family member 2
	EPB41L3	605331	NM_012307.1	0.044	Erythrocyte membrane protein band 4.1-like 3 (suppressor gene?)
	MTUS1	609589	NM_024307.1	0.045	Mitochondrial tumor suppressor 1
Metabolism	HMGCL	246450	NM_000191.1	0.040	3-hydroxymethyl-3-methylglutaryl-Coenzyme A lyase
	UGDH	603370	NM_003359.1	0.041	UDP-glucose dehydrogenase
	CA12	603263	NM_001218.2	0.044	Carbonic anhydrase XII
	CA2	259730	NM_000067.1	0.049	Carbonic anhydrase II
	CA4	114760	NM_000717.2	0.050	Carbonic anhydrase IV
	CA1	114800	NM_001738.1	0.050	Carbonic anhydrase I
	CA7	114770	NM_005182.1	0.050	Carbonic anhydrase VII
	HPGD	601688	U63296.1	0.046	Hydroxyprostaglandin dehydrogenase 15-(NAD)
	FUCA1	230000	NM_000147.1	0.047	Fucosidase, alpha-L-1, tissue
	ACAT1	607809	NM_000019.1	0.048	Acetyl-Coenzyme A acetyltransferase 1
	ADH1C	103730	NM_000669.2	0.048	Alcohol dehydrogenase3 (class I), gamma polypeptide
	AQP8	603750	NM_001169.1	0.050	Aquaporin 8
Cell growth	FAM107A	608295	NM_007177.1	0.040	Family with sequence similarity 107, member A (TU3A)
	EMP1	602333	NM_001423.1	0.047	Epithelial membrane protein 1 (growth arrest)
	BTG1	109580	NM_00173 1.1	0.050	B-cell translocation gene 1, anti-proliferative
	KLF4	602253	NM_004235.1	0.050	Kruppel-like factor 4 (gut)

## References

[B1] Golub TR, Slonim DK, Tamayo P, Huard C, Gaasenbeek M, Mesirov JP, Coller H, Loh ML, Downing JR, Caligiuri MA, Bloomfield CD, Lander ES (1999). Molecular Classification of Cancer: Class Discovery and Class Prediction by Gene Expression Monitoring. Science.

[B2] Tibshirani R, Hastie T, Narasimhan B, Chu G (2002). Diagnosis of multiple cancer types by shrunken centroids of gene expression. Proc Natl Acad Sci.

[B3] Alon U, Barkai N, Notterman DA, Gish K, Ybarra S, Mack D, Levine AJ (1999). Broad patterns of gene expression revealed by clustering analysis of tumor and normal colon tissues probed by oligonucleotide arrays. Proc Natl Acad Sci.

[B4] Ramaswamy S, Tamayo P, Rifkin R, Mukherjee S, Yeang C, Angelo M, Ladd C, Reich M, Latulippe E, Mesirov J, Poggio T, Gerald W, Loda M, Lander E, Golub T (2001). Multiclass cancer diagnosis using tumor gene expression signatures. Proc Natl Acad Sci.

[B5] Rifkin R, Mukherjee S, Tamayo P, Ramaswamy S, Yeang C, Angelo M, Reich M, Poggio T, Lander E, Golub T, Mesirov J (2003). An Analytical Method for Multi-class Molecular Cancer Classification. SIAM Reviews.

[B6] Ambroise C, McLachlan G (2002). Selection bias in gene extraction on the basis of microarray gene-expression data. Proc Natl Acad Sci.

[B7] Simon R, Radmacher M, Dobbin K, McShane L (2003). Pitfalls in the use of DNA Microarray data for diagnostic and prognostic classification. J Natl Cancer Inst.

[B8] Zhang H, Yu C, Singer B, Xiong M (2001). Recursive partitioning for tumor classification with gene expression microarray data. Proc Natl Acad Sci.

[B9] Guyon I, Weston J, Barnhill S, Vapnik V (2002). Gene selection for cancer classification using Support Vector Machines. Machine Learning.

[B10] Vapnik V (1998). Statistical Learning Theory.

[B11] Luntz A, Brailovsky V (1969). On estimation of characters obtained in statistical procedure of recognition. Technicheskaya Kibernetica.

[B12] Mukherjee S, Tamayo P, Rogers S, Rifkin R, Engle A, Campbell C, Golub T, Mesirov J (2003). Estimating Dataset Size Requirements for Classifying DNA Microarray Data. J Comp Biol.

[B13] Radmacher M, McShane L, Simon R (2002). A Paradigm for Class Prediction Using Gene Expression Profiles. J Comp Biol.

[B14] Slonim D, Tamayo P, Mesirov J, Golub T, Lander E (2000). Class Prediction and Discovery Using Gene Expression Data. Proceedings of the Fourth Annual Conference on Computational Molecular Biology (RECOMB).

[B15] Rifkin R, Yeo G, Poggio T, Suykens, Horvath, Basu, Micchelli, Vandewalle (2003). Regularized Least Squares Classification. Advances in Learning Theory: Methods, Model and Applications, NATO Science Series III: Computer and Systems Sciences.

[B16] Ancona N, Maglietta R, D'Addabbo A, Liuni S, Pesole G (2005). Regularized Least Squares Cancer Classifiers from DNA microarray data. BMC-Bioinformatics.

[B17] Good P (1994). Permutation tests: a practical guide to resampling methods for testing hypothesis.

[B18] Nichols T, Holmes A (2001). Nonparametric permutation tests for functional neuroimaging: a primer with examples. Hum Brain Mapp.

[B19] Hastie T, Tibshirani R, Friedman J (2001). The elements of statistical learning Springer series in statistics.

[B20] Kops G, Weaver B, Cleveland D (2005). On the road to cancer: aneuploidy and the mitotic checkpoint. Nat Rev Cancer.

[B21] Cahill D, da Costa L, Carson-Walter E, Kinzler K, Vogelstein B, Lengauer C (1999). Characterization of MAD2B and other mitotic spindle checkpoint genes. Genomics.

[B22] Yuan B, Xu Y, Woo J, Wang Y, Bae Y, Yoon D, Wersto R, Tully E, Wilsbach K, Gabrielson E (2006). Increased expression of mitotic checkpoint genes in breast cancer cells with chromosomal instability. Clin Cancer Res.

[B23] Shapira M, Ben-Izhak O, Bishara B, Futerman B, Minkov I, Krausz M, M MP, Hershko D (2004). Alterations in the expression of the cell cycle regulatory protein cyclin kinase subunit 1 in colorectal carcinoma. Cancer.

[B24] Li M, Lin Y, Hasegawa S, Shimokawa T, Murata K, Kameyama M, Ishikawa O, Katagiri T, Tsunoda T, Nakamura Y, Furukawa Y (2004). Genes associated with liver metastasis of colon cancer, identified by genome-wide cDNA microarray. Int J Oncol.

[B25] Tsai Y, Chang H, Chuang L, Hung W (2005). RNA silencing of Cks1 induced G2/M arrest and apoptosis in human lung cancer cells. IUBMB Life.

[B26] Wang I, Chen Y, Hughes D, Petrovic V, Major M, Park H, Tan Y, Ackerson T, Costa R (2005). Forkhead box Ml regulates the transcriptional network of genes essential for mitotic progression and genes encoding the SCF (Skp2-Cks1) ubiquitin ligase. Mol Cell Biol.

[B27] Zhou H, Kuang J, Zhong L, Kuo W, Gray J, Sahin A, Brinkley B, Sen S (1998). Tumour amplified kinase STK15/BTAK induces centrosome amplification, aneuploidy, and transformation. Nat Genet.

[B28] Hayes G, Carrigan P, Beck A, Miller L (2006). Targeting the RNA splicing machinery as a novel treatment strategy for pancreatic carcinoma. Cancer Res.

[B29] Kimura K, Cuvier O, Hirano T (2001). Chromosome condensation by a human condensin complex in Xenopus egg extracts. J Biol Chem.

[B30] Geiman T, Sankpal U, Robertson A, Chen Y, Mazumdar M, Heale J, Schmiesing J, Kim W, Yokomori K, Zhao Y, Robertson K (2004). Isolation and characterization of a novel DNA methyltransferase complex linking DNMT3B with components of the mitotic chromosome condensation machinery. Nucleic Acids Res.

[B31] Adamson E, Minchiotti G, Salomon D (2002). Cripto: a tumor growth factor and more. J Cell Physiol.

[B32] Fujita M, Furukawa Y, Tsunoda T, Tanaka T, Ogawa M, Nakamura Y (2001). Up-regulation of the ectodermal-neural cortex 1 (ENC1) gene, a downstream target of the beta-catenin/T-cell factor complex, in colorectal carcinomas. Cancer Res.

[B33] Blache P, van de Wetering M, Duluc I, Domon C, Berta P, Freund J, Clevers H, Jay P (2004). SOX9 is an intestine crypt transcription factor, is regulated by the Wnt pathway, and represses the CDX2 and MUC2 genes. J Cell Biol.

[B34] Kau T, Way J, Silver P (2004). Nuclear transport and cancer: from mechanism to intervention. Nat Rev Cancer.

[B35] Hagenbuch B, Meier P (2004). Organic anion transporting polypeptides of the OATP/SLC21 family: phylogenetic classification as OATP/SLCO superfamily, new nomenclature and molecular/functional properties. Pflugers Arch.

[B36] Yamana K, Bilim V, Hara N, Kasahara T, Itoi T, Maruyama R, Nishiyama T, Takahashi K, Tomita Y (2005). Prognostic impact of FAS/CD95/APO-1 in urothelial cancers: decreased expression of Fas is associated with disease progression. Br J Cancer.

[B37] Chen Y, Knosel T, Kristiansen G, Pietas A, Garber M, Matsuhashi S, Ozaki I, Petersen I (2003). Loss of PDCD4 expression in human lung cancer correlates with tumour progression and prognosis. J Pathol.

[B38] Yang H, Matthews C, Clair T, Wang Q, Baker A, Li C, Tan T, Colburn N (2006). Tumorigenesis suppressor Pdcd4 down-regulates mitogen-activated protein kinase kinase kinase kinase 1 expression to suppress colon carcinoma cell invasion. Mol Cell Biol.

[B39] Lusis E, Watson M, Chicoine M, Lyman M, Roerig P, Reifenberger G, Gutmann D, Perry A (2005). Integrative genomic analysis identifies NDRG2 as a candidate tumour suppressor gene frequently inactivated in clinically aggressive meningioma. Cancer Res.

[B40] Kittiniyom K, Mastronardi M, Roemer M, Wells W, Greenberg E, Titus-Ernstoff L, Newsham I (2004). Allele-specific loss of heterozygosity at the DAL-1/4.1B (EPB41L3) tumour-suppressor gene locus in the absence of mutation. Genes Chromosomes Cancer.

[B41] Seibold S, Rudroff C, Weber M, Galle J, Wanner C, Marx M (2003). Identification of a new tumor suppressor gene located at chromosome 8p21.3–22. FASEB J.

[B42] Wang L, Darling J, Zhang J, Liu W, Qian J, Bostwick D, Hartmann L, Jenkins R, Bardenhauer W, Schutte J, Opalka B, Smith D (2000). Loss of expression of the DRR 1 gene at chromosomal segment 3p21.1 in renal cell carcinoma. Genes Chromosomes Cancer.

[B43] Zhao W, Hisamuddin I, Nandan M, Babbin B, Lamb N, Yang V (2004). Identification of Kruppel-like factor 4 as a potential tumor suppressor gene in colorectal cancer. Oncogene.

[B44] Wei D, Gong W, Kanai M, Schlunk C, Wang L, Yao J, Wu T, Huang S, Xie K (2005). Drastic down-regulation of Kruppel-like factor 4 expression is critical in human gastric cancer development and progression. Cancer Res.

[B45] Furlanello C, Serafini M, Merler S, Jurman G (2003). Entropy-based gene ranking without selection bias for the predictive classification of microarray data. BMC-Bioinformatics.

